# CRISPR/dCas9-mediated tuning of *DMPK* transcription reveals a quantitative relationship between toxic repeat RNA expression and MBNL1 activity in myotonic dystrophy

**DOI:** 10.1093/hmg/ddag079

**Published:** 2026-08-21

**Authors:** Lise Ripken, Thomas D Hoekman, Marieke Willemse, Maeve L Troost, Angelique N Kenyon, Walther J A A van den Broek, Derick G Wansink

**Affiliations:** Radboud University Medical Center, Radboud Institute for Medical Innovation, Department of Medical BioSciences, 6525 GA, Nijmegen, The Netherlands; Radboud University Medical Center, Radboud Institute for Medical Innovation, Department of Medical BioSciences, 6525 GA, Nijmegen, The Netherlands; Radboud University Medical Center, Radboud Institute for Medical Innovation, Department of Medical BioSciences, 6525 GA, Nijmegen, The Netherlands; Radboud University Medical Center, Radboud Institute for Medical Innovation, Department of Medical BioSciences, 6525 GA, Nijmegen, The Netherlands; Radboud University Medical Center, Radboud Institute for Medical Innovation, Department of Medical BioSciences, 6525 GA, Nijmegen, The Netherlands; Radboud University Medical Center, Radboud Institute for Medical Innovation, Department of Medical BioSciences, 6525 GA, Nijmegen, The Netherlands; Radboud University Medical Center, Radboud Institute for Medical Innovation, Department of Medical BioSciences, 6525 GA, Nijmegen, The Netherlands

**Keywords:** CRISPR/dCas9, DMPK, MBNL1, transcription modulation, triplet repeat

## Abstract

Myotonic dystrophy type 1 (DM1) is caused by (CUG)n-expanded *DMPK* transcripts that sequester the splicing factor MBNL1 in the nucleus, resulting in widespread splicing abnormalities. Although significant progress has been made in understanding DM1 pathogenesis, the contribution of *DMPK* transcript levels to disease severity, and the variability of these levels across cell types, tissues, and patients, remains poorly understood. To investigate this in a quantitative manner, we developed isogenic human immortalized myoblast models with inducible modulation of *DMPK* RNA levels using CRISPR activation (CRISPRa) and interference (CRISPRi) guided by synthetic sgRNAs. CRISPRa elevated *DMPK* RNA levels by more than three-fold, intensifying MBNL1-dependent splicing defects. In contrast, CRISPRi reduced *DMPK* RNA expression by approximately 80%, partially rescuing splicing abnormalities. These changes were validated by visualizing (CUG)n foci using RNA FISH. Lowering *DMPK* transcript levels increased the availability of free nucleoplasmic MBNL1, whereas upregulation further depleted MBNL1, reinforcing the central role of MBNL1 sequestration in repeat RNA toxicity. Our findings demonstrate that expanded *DMPK* transcript levels modulate free MBNL1 concentration and alternative splicing in a dose-dependent manner, underscoring the central role of repeat RNA expression in DM1 pathogenesis. These models provide a powerful platform for dissecting variability in *DMPK* expression and for defining the therapeutic thresholds required for effective *DMPK* knockdown, thereby offering critical insights for the design and evaluation of *DMPK* and *MBNL1*-directed therapeutic strategies.

## Introduction

The neuromuscular disorder myotonic dystrophy type 1 (DM1) is caused by a dominantly inherited (CTG)n triplet repeat expansion in the 3’ untranslated region of the DM1 protein kinase (*DMPK*) gene [1–3]. While the expansion is harmless below 37 triplets, those exceeding this threshold become unstable and can expand to thousands of triplets both somatically and in the germline. Patients suffer from multiorgan symptoms ranging from cataracts, typically seen in late-onset DM1, to congenital intellectual impairment and muscle-related symptoms like myotonia and weakness. Progressively longer repeat expansions in successive generations with DM1 correlate with earlier onset and increased severity, a phenomenon known as clinical anticipation [4, 5].

The repeat expansion does not alter the DMPK protein sequence but instead causes disease through a toxic RNA gain-of-function mechanism [6–8]. Analyses of DM1 muscle and brain tissue have revealed profound transcriptomic changes, most notably a shift from adult to fetal RNA splicing patterns [9–11]. Commonly misspliced genes linked to DM1 symptoms encompass *BIN1* (muscle weakness), *SCN5A* (cardiac arrhythmias) and *CLCN1* (myotonia) [10, 12–14]. Imbalance in alternative splicing is caused mainly by sequestration of the RNA-binding protein muscleblind-like splicing regulator 1 (MBNL1) by the (CUG)n repeat in expanded *DMPK* transcripts [15–17]. The resulting RNA-protein aggregates can be microscopically visualized and quantified as so-called RNA foci in the cell nucleus, using a combination of fluorescence *in situ* hybridization (FISH) and immunofluorescence staining [18, 19]. In the absence of functional MBNL1 protein, as mimicked in *MBNL1* and *MBNL2* deficient cells or in the presence of a MBNL1 decoy, these RNA foci disintegrate and expanded *DMPK* transcripts may be degraded [20, 21]. The aggregative interplay between individual *DMPK* transcripts and MBNL1 proteins within nuclear foci, along with their spatial and temporal dynamics, is a key research object in DM1 pathogenesis and a promising target for therapy development.

To clarify the relationship between (CUG)n-expanded *DMPK* transcripts and symptom severity in DM1 patients, the field focused on understanding the correlation between residual MBNL1 activity and the extent of aberrant alternative splicing. *In vitro* modulation of MBNL1 protein levels using an inducible promoter has revealed a clear dose-dependent relationship between MBNL1 activity and the inclusion or exclusion of selected DM1-typical splicing events [22]. By extrapolating this MBNL1-splicing relationship to patient tissues, an inferred MBNL1 activity level was estimated based on the degree of missplicing. Further, the extent of splicing abnormalities correlates with disease severity and may even serve as a prognostic marker for the DM1 phenotype in patients [23, 24]. It currently remains unclear, however, how these functional readouts relate to *DMPK* expression itself, a quantifiable measure of expanded *DMPK* transcript abundance and thus of disease load at the RNA level.

This relationship is complicated by the marked variability in the length of pathogenic (CTG)n repeats. Repeat length differs not only between patients, but also across tissues and even between individual cells within the same tissue as a consequence of ongoing somatic instability. The severity of DM1 manifestation is dependent on the repeat length, but the clinical impact of a certain repeat length is highly heterogenous and difficult to predict [5, 25, 26]. Moreover, repeat lengths are typically measured in blood, but are much longer in affected tissues like muscle [27, 28]. We hypothesize that the disease burden of expanded *DMPK* genes is dictated by a combination of cell type, (CTG)n expansion length, and abundance of expanded transcripts within individual cells [24, 29–31].

Varying expansion lengths in a panel of isogenic DM1 myoblasts have indeed shown that repeat length correlates with DM1-typical missplicing [32]. Building on this work, we sought to develop an isogenic model based on the same immortalized cell line in which *DMPK* RNA expression could be modulated without altering the repeat length, to study its effects on MBNL1 availability and alternative splicing. We employed catalytically inactive Cas9 (dCas9) fused to either a transcription activator (CRISPRa) or a repressor (CRISPRi) to modulate *DMPK* transcript expression, and thus (CUG)n expanded RNA abundance, in an inducible manner [33, 34]. This system enabled us to tune *DMPK* transcript levels without the use of therapeutic agents. RNA quantification combined with advanced microscopy revealed a clear, dose-dependent relationship between *DMPK* transcript abundance, nuclear MBNL1 availability and alternative splicing regulation.

## Results

To reveal the relation between the synthesis and steady-state expression of expanded *DMPK* RNA and downstream pathological processes, *DMPK* transcription was up- and downregulated under controlled conditions using established CRISPRa/i systems. We utilized *TERT*- and *CDK4-*immortalized DM1 myoblasts bearing a healthy allele with 13 and an expanded allele with a modal length of 2,900 triplets [32]. These DM1 myoblasts have been previously characterized and exhibit a profound congenital DM1 phenotype with aberrant alternative splicing, hypermethylated DM1 locus, decreased nuclear MBNL1 protein concentration and impaired myogenic capacity [35, 36]. As a control reference line, we took along our previously generated, isogenic, gene-edited DM∆ cell line in which the repeat expansion was excised using a dual Cas9 approach [37].

### CRISPRa-mediated upregulation of *DMPK* expression results in an increased number of RNA foci

First, we investigated whether known DM1 hallmarks could be further aggravated by increasing *DMPK* expression and therefore expanded (CUG)n dose within the cell. To achieve this, we lentivirally transduced DM1 myoblasts and isogenic DM∆ myoblasts lacking the expanded repeat as a control, generating stable cell lines that constitutively express dCas9 coupled to a VPR domain, consisting of VP64, p65 and Rta transcription activators, referred to as DM1^dCas9VPR^ and DM∆^dCas9VPR^ [38]. Multiple CRISPRa single guide RNAs (sgRNAs) were designed to bind maximally ~500 bp upstream of the *DMPK* transcription start site in line with the optimal proposed binding sites in CRISPRa to achieve transcriptional upregulation [34]. SgRNAs CRa 1, 4 and 5 were selected for efficiency testing based on their highest on-target and lowest off-target predictions (Fig. 1A; Supplementary Material, Fig. S1).

**Figure 1:**
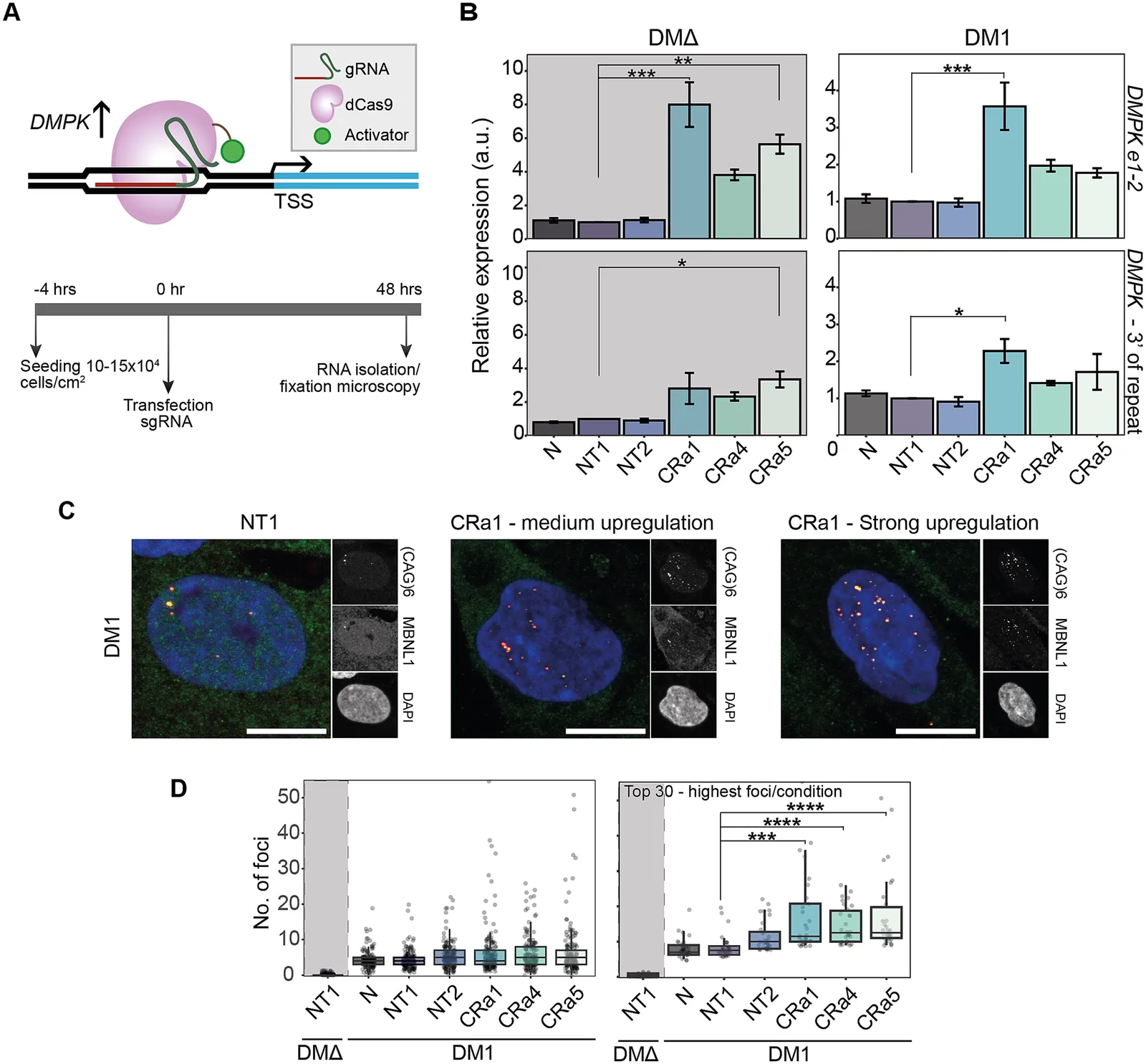
Inducible upregulation of *DMPK* RNA expression via CRISPRa. A) Schematic representation of the CRISPRa technology and experimental set up. dCas9 coupled to activating transcription factor VPR was targeted upstream of the transcription start site (TSS) of *DMPK* (gRNA sequences in Supplementary Table 1). Myoblasts, either DM1^dCas9VPR^ or isogenic, dual Cas9 corrected DM∆^dCas9VPR^, were seeded on culture plates and lipofectamine-mediated transfection of synthetic sgRNAs (2 and 10 nM for DM∆ and DM1, resp.) was performed. After 48 hours, RNA was isolated and cells were fixed for microscopy. B) *DMPK* RNA levels were assessed using amplicons spanning the exon 1-2 junction, or located in exon 15, 3’ of the repeat expansion, following CRISPRa with targeting sgRNAs (CRa1, 4 and 5), control sgRNAs (NT1 and 2), or no gRNA (N). One-way ANOVA followed by Tukey’s multiple comparisons testing was performed to assess significance. Significance is shown compared to NT1. n=3 independent experiments, with two wells per condition per experiment as technical duplicate. Error bars depict SEM. C) At 10 nM sgRNA, foci were visualized using FISH against (CUG)n-expanded RNA (red), immunofluorescent labelling of MBNL1 (green) and nuclear staining (blue). Three typical example nuclei are shown. Scale bar is 10 μm. D) The number of nuclear foci per cell was counted using automated analysis. Each datapoint represents one cell. The top 30 nuclei with highest foci number are highlighted in the right panel. A Kruskal-Wallis followed by a Dunn’s test for multiple testing was used to compare each condition. Significance is shown towards NT1. n=3 independent experiments, per n at least 35 nuclei in 5-10 images were captured. ^*^P < .05, ^**^P < .01, ^***^P < .001, ^****^P < .0001.

DM1^dCas9VPR^ and DM∆^dCas9VPR^ cells were seeded at low confluency and transfected with a presumed near-100% efficiency using synthetic sgRNAs CRa1, 4 or 5 and, as controls, no sgRNA (N) and two non-targeting sgRNAs (NT1 and NT2). All analyses were performed 48 hours after transfection in a subconfluent cell culture to allow monitoring of downstream effects (Fig. 1A). *DMPK* RNA expression was assessed via RT-qPCR using two amplicons (spanning exon 1-2 and in exon 15, 3’ of the repeat) to ensure an effect of the CRISPRa tool towards the full-length *DMPK* transcript. Upon transfection of 10 nM CRa sgRNAs in DM1^dCas9VPR^ myoblasts, *DMPK* transcripts were upregulated an average 2-3.5-fold compared to NT1 as assessed at exon 1-2 (Fig. 1B). The effect on the downstream repeat-containing exon was slightly lower and displayed a significant average increase of 1.5-2.5-fold. The reference line DM∆^dCas9VPR^, transfected with 2 nM synthetic sgRNA, also demonstrated efficient upregulation of *DMPK*, with levels comparable to and in some cases exceeding those observed in DM1^dCas9VPR^, thereby serving as a proper control for *DMPK* upregulation. We consistently observed that *DMPK* RNA levels in DM1 cells did not reach those in DM∆ cells, even when the sgRNA dose was increased (Supplementary Material, Fig. S2A). This suggests a toxic effect on cells when the expression level of (CUG)n-containing transcripts becomes too high.

Next, we examined the effect of increased (CUG)n expanded transcript levels in DM1^dCas9VPR^ cells on the presence of nuclear RNA foci, a key DM1 disease hallmark. FISH performed with a (CAG)6 probe combined with staining for MBNL1 protein was used to visualize RNA foci. For each targeting sgRNA, myoblasts were observed in which the number of nuclear foci greatly exceeded the average number of foci found under control conditions (Fig. 1C; 10 nM sgRNA). CRISPRa had a heterogenous effect across the cell population, but treatment with CRa1, 4 and 5 led to a marked increase in foci numbers (NT1 averaged 4.4 foci/nucleus; CRa1, 4, 5: 6.2-6.6), significantly so when focusing on the top 30 cells with the highest foci counts (NT1 averaged 8.6 foci/nucleus; CRa1, 4, 5: 14.6-17.5), reaching up to several dozen per nucleus (Fig. 1D). We have no explanation for the increased number of foci caused by NT2. As expected, DM∆^dCas9VPR^ did not show any RNA foci since the expanded repeat-was absent in these cells. In conclusion, the *DMPK* promoter is responsive to CRISPRa-mediated activation, leading to an upregulation of *DMPK* RNA expression and a concomitant rise in RNA foci number.

### Efficient downregulation of *DMPK* RNA expression by CRISPRi reduces the number of RNA foci

In addition to transcriptional activation, we employed CRISPRi, an opposing approach to CRISPRa, using dCas9 fused to the transcriptional repressor Krüppel-associated box (dCas9-KRAB), to reduce *DMPK* RNA expression and mimic the potential effects of RNA elimination therapies currently in clinical trials. Parental DM1 and DM∆ lines were lentivirally stabilized for dCas9-KRAB (DM1^dCas9KRAB^ and DM∆^dCas9KRAB^, respectively). Interference sgRNAs were designed to target dCas9-KRAB protein downstream of the *DMPK* transcription start site and sterically hinder RNA polymerase II progression (Fig. 2A; Supplementary Material, Fig. S1). Keeping the protocol similar to the CRISPRa experiments, myoblasts were transfected using targeting CRISPRi sgRNAs (CRi3, 4 or 5) or control conditions, and analyses were performed after 48 hours under subconfluent culture conditions. Using all three CRi sgRNAs, mean *DMPK* expression was lowered by 74-84% for exon 1-2 in DM1^dCas9KRAB^ and control DM∆ ^dCas9KRAB^ cell lines. Knockdown efficiencies for the exon 15 amplicon were comparable, though slightly lower, ranging from 59-75% (Fig. 2B).

**Figure 2:**
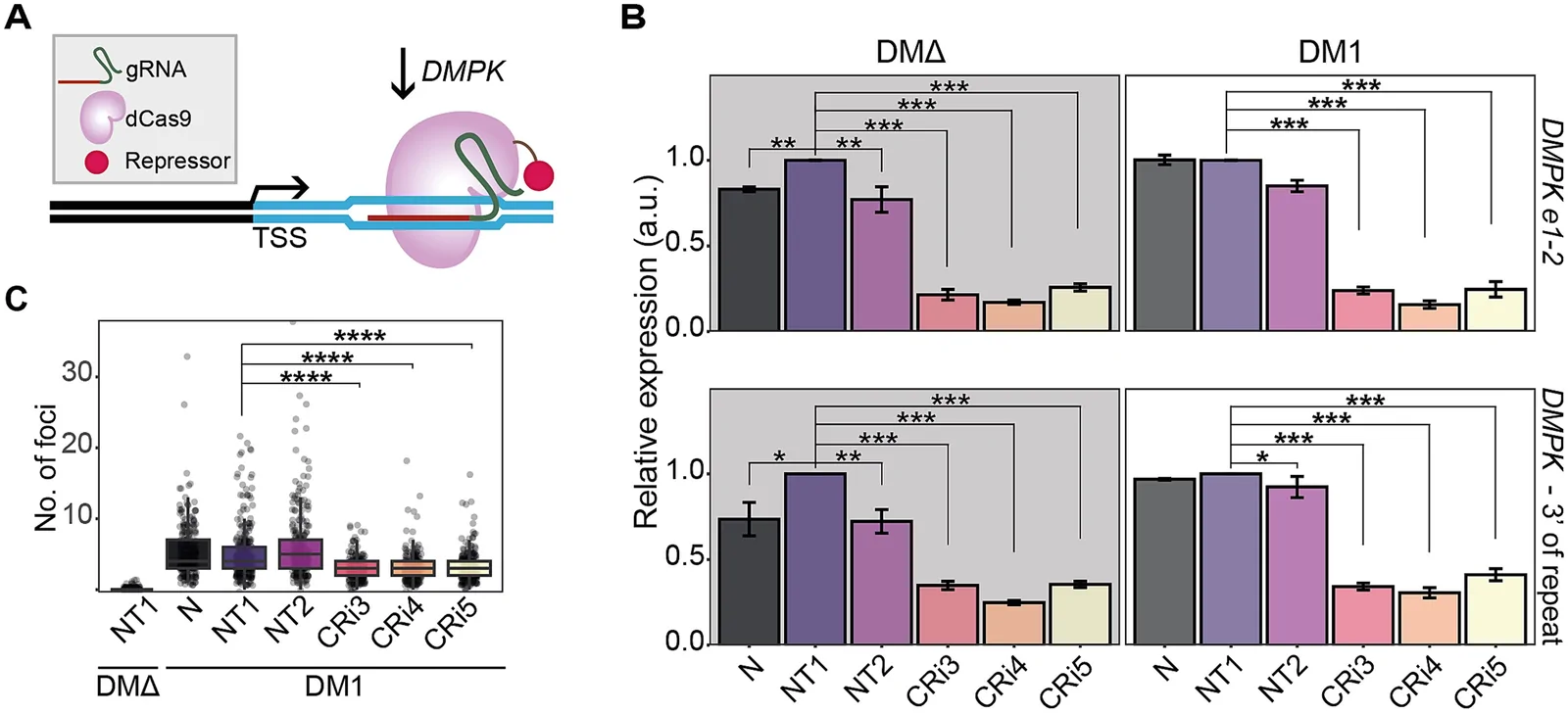
CRISPRi targeted at *DMPK* gene represses transcription and decreases foci number. A) Schematic representation of CRISPRi technology. dCas9 coupled to repressing transcription factor, KRAB, was targeted downstream of the transcription start site of *DMPK*. B) DM1 or DM∆ myoblasts, stabilized for dCas9-KRAB, were seeded on culture plates and transfected with synthetic sgRNAs (5 nM). RNA isolation and fixation for microscopy were performed after 48 hours. Three *DMPK* targeting sgRNAs (CRi 3, 4 and 5) were used, while two non-targeting sgRNAs (NT1 and 2) and no sgRNA (N) were included as controls. *DMPK* RNA levels were assessed by RT-qPCR using amplicons spanning the exon 1-2 junction, or located in exon 15, 3’ of the repeat expansion. One-way ANOVA followed by Tukey multiple comparisons testing was performed to assess significance. Significance is shown compared to NT1. n=3 independent experiments, with two wells per condition per experiment as technical duplicates. Error bars depict SEM. C) In parallel, foci were visualized using FISH against (CUG)n RNA, immunofluorescent labelling of MBNL1 and nuclear staining. Confocal images were analyzed using automated macro scripts to count foci numbers per single nuclei. Data points represent the number of foci per single nucleus. A Kruskal-Wallis followed by a Dunn’s test for multiple testing was used to compare each condition. Significance is shown towards NT1. n=3 independent experiments, per n at least 45 nuclei in 5-10 images were analyzed per condition. ^*^P < .05, ^**^P < .01, ^***^P < .001.

RNA FISH, followed by confocal microscopy and automated quantification, confirmed a significant decrease of over 50% in RNA foci number in DM1^dCas9KRAB^ cells (Fig. 2C), complementing the RT-qPCR observations. Thus, CRISPRi efficiently reduced the number of (CUG)n-containing *DMPK* transcripts, which in turn led to a decrease in RNA foci in our DM1 cell model.

Altogether, dCas9-mediated tuning of *DMPK* expression proved to be a feasible strategy to modify steady-state *DMPK* RNA levels and foci number in myoblasts, thereby enabling the investigation of how changes in transcript abundance influence the severity of downstream pathogenic effects, particularly the hallmark DM1 phenotype of aberrant alternative splicing.

### Dose-dependent effects of expanded *DMPK* RNA on DM1-associated splicing events

One of the most predictive DM1 biomarkers for disease severity to date is misregulated alternative splicing. We have carefully chosen three cassette exons for which a clear baseline difference was observed in our DM1 and rescued DM∆ cell lines. Excessive *MBNL1 e5* inclusion is commonly reported in DM1 literature. The ratio exon inclusion over exclusion, or percentage spliced in (Ψ), is typically low for this alternative splice event, and shifts towards higher inclusion due to a shortage of MBNL1 protein in DM1 patient cells. Additionally, *CLASP1 e19* (preferred exclusion in DM1) and *NFIX e7* (preferred inclusion in DM1) were selected because of their statistically high predictability for DM1 severity and moderate sensitivity for MBNL1-mediated splicing [22, 24]. RT-PCR followed by capillary gel electrophoresis was performed to determine Ψ for the designated cassette exons in the DM1 and control samples described above.

Upregulation of expanded *DMPK* RNA in DM1^dCas9VPR^ myoblasts resulted in increased Ψ levels for *MBNL1 e5*, indicating a more severely misspliced transcriptome (Ψ levels increased from 0.48 (NT1) to 0.62-0.63 (CRa1, 4 and 5); ∆Ψ of max. 0.15), (Fig. 3A). Interestingly, the other two alternative splicing markers examined, *NFIX* and *CLASP1*, were not noticeably affected by elevated *DMPK* levels. This observation suggests that either the degree of *DMPK* expanded RNA upregulation was insufficient to further disrupt splicing of these targets, or that these splicing events were already maximally misregulated. Since all three CRa sgRNAs induced similar levels of missplicing regardless of modest differences in *DMPK* RNA expression, our data support the latter explanation. Notably, upregulation of the *DMPK* gene *per se* cannot account for the observed changes, since Ψ levels in myoblasts lacking the repeat expansion (DM∆^dCas9VPR^) remained unaffected.

**Figure 3:**
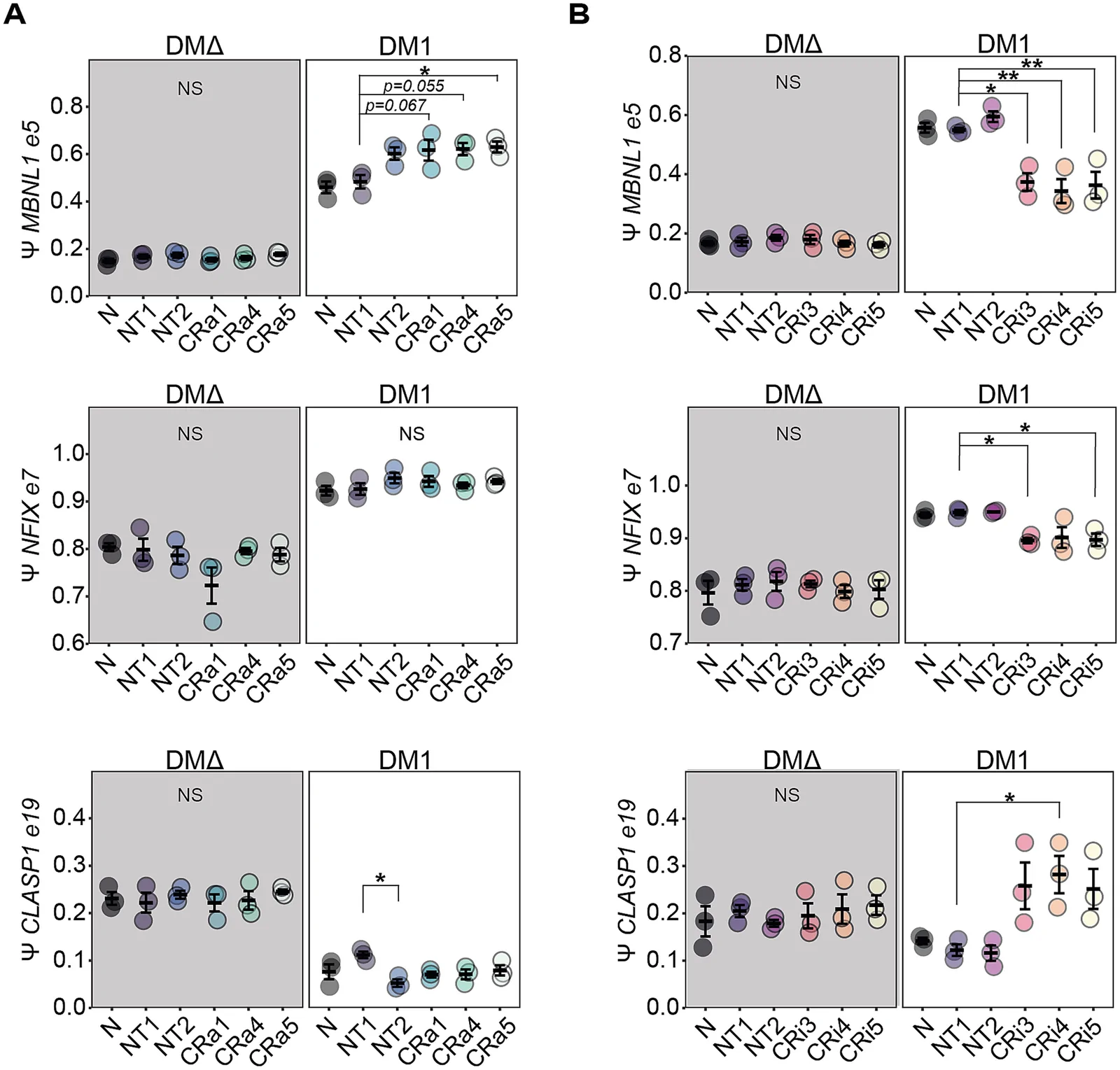
Aberrant DM1 splicing events are sensitive to varying *DMPK* RNA levels. Ψ levels for three meaningful DM1 splicing events were assessed in DM1^dCas9KRAB^ and DM∆^dCas9KRAB^ treated with CRISPRa (A) and CRISPRi (B) for 48 hours by RT-PCR followed by capillary gel electrophoresis. n=3 independent experiments, with two wells per condition per experiment as technical duplicate; data points represent the average of the two technical replicates. One-way ANOVA with Tukey multiple comparisons testing was performed per cell type to compare treated samples to NT1. Error bars represent SEM. ^*^P < .05, ^**^P < .01.

In contrast, CRISPRi-mediated inhibition of *DMPK* led to significant changes across all three alternative splicing events examined (Fig. 3B). An approximately 80% reduction in *DMPK* expression partially rescued Ψ levels for *MBNL1* (Ψ levels decreased from 0.55 (NT1) to 0.34-0.37 (CRi3, 4 and 5); ∆Ψ of max. 0.21), and *NFIX* (Ψ levels decreased from 0.95 (NT1) to 0.90 (CRi3, 4 and 5); ∆Ψ of max. 0.05) , bringing them to about halfway between the DM1 and DM∆ states. Remarkably, *CLASP1* exhibited a full reversal, or even an overcompensatory phenotype, upon knockdown with each of the CRi sgRNAs (Ψ levels increased from 0.12 (NT1) to 0.25-0.28 (CRi3, 4 and 5); ∆Ψ of max. 0.16). Importantly, *DMPK* downregulation in the DM∆^dCas9KRAB^ cell model did not alter splicing of any of the analyzed events, providing and essential control and indicating that reduced expanded *DMPK* levels ameliorate the splicing phenotype in DM1 myoblasts.

As additional evidence supporting the quantitative relationship between *DMPK* RNA levels and downstream missplicing, we titrated sgRNA concentrations in both CRISPRa and CRISPRi stable cell lines. In CRISPRa lines, increasing the concentration of sgRNA CRa1, resulted in a dose-dependent increase in *DMPK* RNA levels although a plateau was reached relatively early (Supplementary Material, Fig. S2A). While 1 nM led to a modest elevation and 5 nM to a more pronounced increase, concentrations higher than 5 nM (10-15 nM) produced similar or slightly lower expression levels, suggesting a saturation or toxic effect. For *MBNL1*, but not for *NFIX* and *CLASP1*, a clear dose-response relationship was observed between CRa1 sgRNA and the extent of missplicing, consistent with earlier findings (Supplementary Material, Fig. S2B). In CRISPRi-treated cells, we observed a robust concentration-dependent downregulation of *DMPK* RNA with increasing CRi4 sgRNA concentrations (Supplementary Material, Fig. S3A). In parallel, alternative splicing showed a consistent dose-response trend: *MBNL1* and *NFIX* responded primarily at lower CRa1 sgRNA concentrations, whereas *CLASP1* showed changes predominantly at higher sgRNA concentrations (Supplementary Material, Fig. S3B), illustrating different correction sensitivities to reduced expanded *DMPK* expression. Altogether, these findings demonstrate that the level of (CUG)n-expanded *DMPK* RNA directly correlates with DM1-characteristic missplicing, highlighting repeat RNA abundance as a key factor in the pathogenic burden of (CUG)n-expanded transcripts.

### *DMPK* RNA expression determines nuclear MBNL1 protein localization

Aberrant alternative splicing in DM1 is thought to result from the formation of (CUG)n-RNA MBNL1 protein aggregates, which sequester MBNL1 and lead to its functional depletion in the nucleus. Given the pronounced impact of modulating *DMPK* RNA levels on alternative splicing in our CRISPRa/i models, we investigated whether this effect could indeed be attributed to changes in free nuclear MBNL1 protein abundance. To quantify MBNL1, we performed immunofluorescent labelling and confocal microscopy of myoblasts (DM1^dCas9VPR^/DM∆^dCas9VPR^ and DM1^dCas9KRAB^/DM∆ ^dCas9KRAB^), transfected with CRa and CRi sgRNAs as described above, followed by fully automated image analysis. This analysis measured nucleoplasmic MBNL1 protein levels by subtracting the signal of MBNL1 sequestered in nuclear foci from the total nuclear MBNL1 signal.

Elevated *DMPK* RNA levels induced by CRISPRa, along with an increase in nuclear foci, were associated with reduced levels of free nucleoplasmic MBNL1 protein (decrease of 10-18% (to 82-90% of NT1), particularly in the top 30 cells per condition exhibiting the highest number of foci per nucleus (reduction of 20-27%) (Fig. 4A and B; Note that, again, for unknown reasons NT2 behaved differently.). Myoblasts with a high number of foci were nearly devoid of free nucleoplasmic MBNL1 protein, with images showing MBNL1 predominantly localized to RNA foci. Conversely, CRISPRi treatment led to a reduction in foci, releasing MBNL1 and resulting in higher levels of free nucleoplasmic MBNL1 in DM1^dCas9KRAB^ compared to DM1 control conditions, and similar to levels observed in DM∆ cells (increase of 44-55%) (Fig. 4C-D).

**Figure 4:**
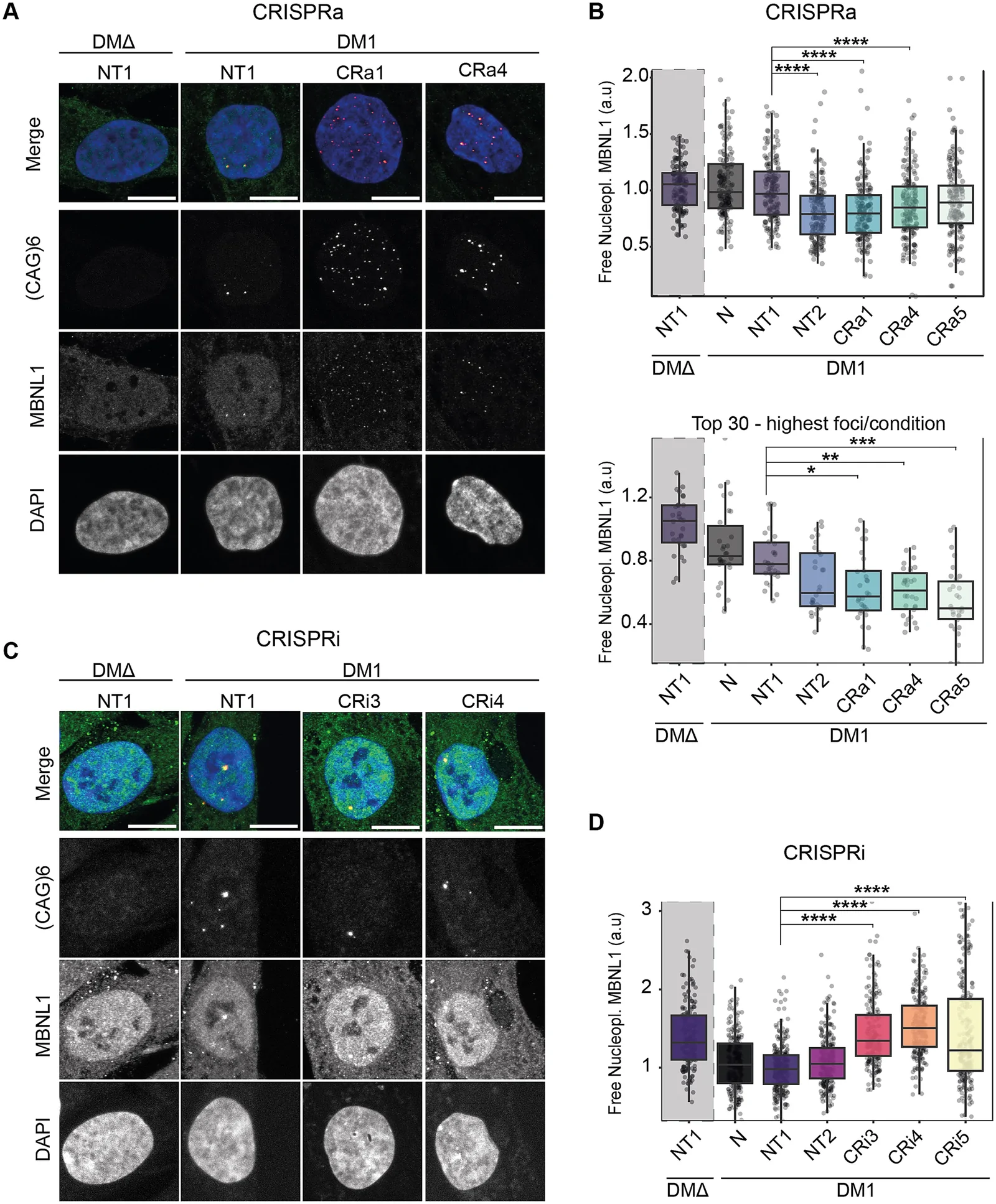
Quantification of free nucleoplasmic MBNL1 following CRISPRa or CRISPRi treatment. DM1 and DM∆ myoblasts were treated for 48 hours with CRISPRa (A-B) or CRISPRi (C-D) using the indicated sgRNAs, then fixed and stained with a (CAG)6 probe (red), followed by immunofluorescent labelling of MBNL1 (green) and a nuclear counterstain (blue). Z-stack images were acquired using confocal microscopy. Representative maximum intensity Z-projections are shown for selected conditions. Scale bar is 10 μm. (B/D) Free nucleoplasmic MBNL1 signal was quantified by subtracting the MBNL1 signal within RNA foci (mask created by (CAG)6 FISH) from the total nucleoplasmic MBNL1 signal (mask created by nuclear stain). Normalization was performed on DM1 NT1 per replicate. Each data point represents one nucleus. A Kruskal-Wallis followed by a Dunn’s test for multiple testing was used to compare each condition. n=3 independent experiments, per n at least 35 nuclei in 5-10 images were analyzed per condition. ^*^P < .05, ^**^P < .01, ^***^P < .001, ^****^P < .0001.

Finally, to further dissect the relationship between *DMPK* RNA expression and MBNL1 availability, we examined whether this coupling could be resolved at the single-cell level using a complementary analytical approach. Given that *DMPK* RNA expression levels correlate with the number of nuclear foci in our CRISPRi and CRISPRa models, we investigated whether a dose-dependent relationship between *DMPK* RNA and MBNL1 protein levels could be observed on a per-cell basis. In this context, each myoblast was considered an individual dosing entity. Conceptually, a lower number of nuclear foci would be expected to result in reduced sequestration of MBNL1 protein, thereby increasing the pool of free nucleoplasmic MBNL1 protein.

To test this hypothesis, DM1 nuclei measured in the above-mentioned experiments were categorized according to their foci count, after which mean levels of free nucleoplasmic MBNL1 were quantified for each category. Clustering analysis across CRISPRi and CRISPRa conditions revealed two distinct groups: one comprising CRISPRi-treated samples, and another encompassing control and CRISPRa samples (Fig. 5). Within the control and CRISPRa group, a clear gradient was observed, whereby nuclei with fewer foci exhibited higher levels of MBNL1 protein, consistent with dose-dependent sequestration. Notably, this gradient was less pronounced in the CRISPRa samples. In contrast, but as expected, CRISPRi-mediated reduction of *DMPK* expression led to a substantial increase in nucleoplasmic MBNL1 protein levels. Notably, this increase was largely independent of the residual number of nuclear RNA foci, suggesting that under conditions of reduced *DMPK* transcription, the relationship between RNA foci number or composition and overall expanded *DMPK* RNA levels is altered in this subset of cells.

**Figure 5:**
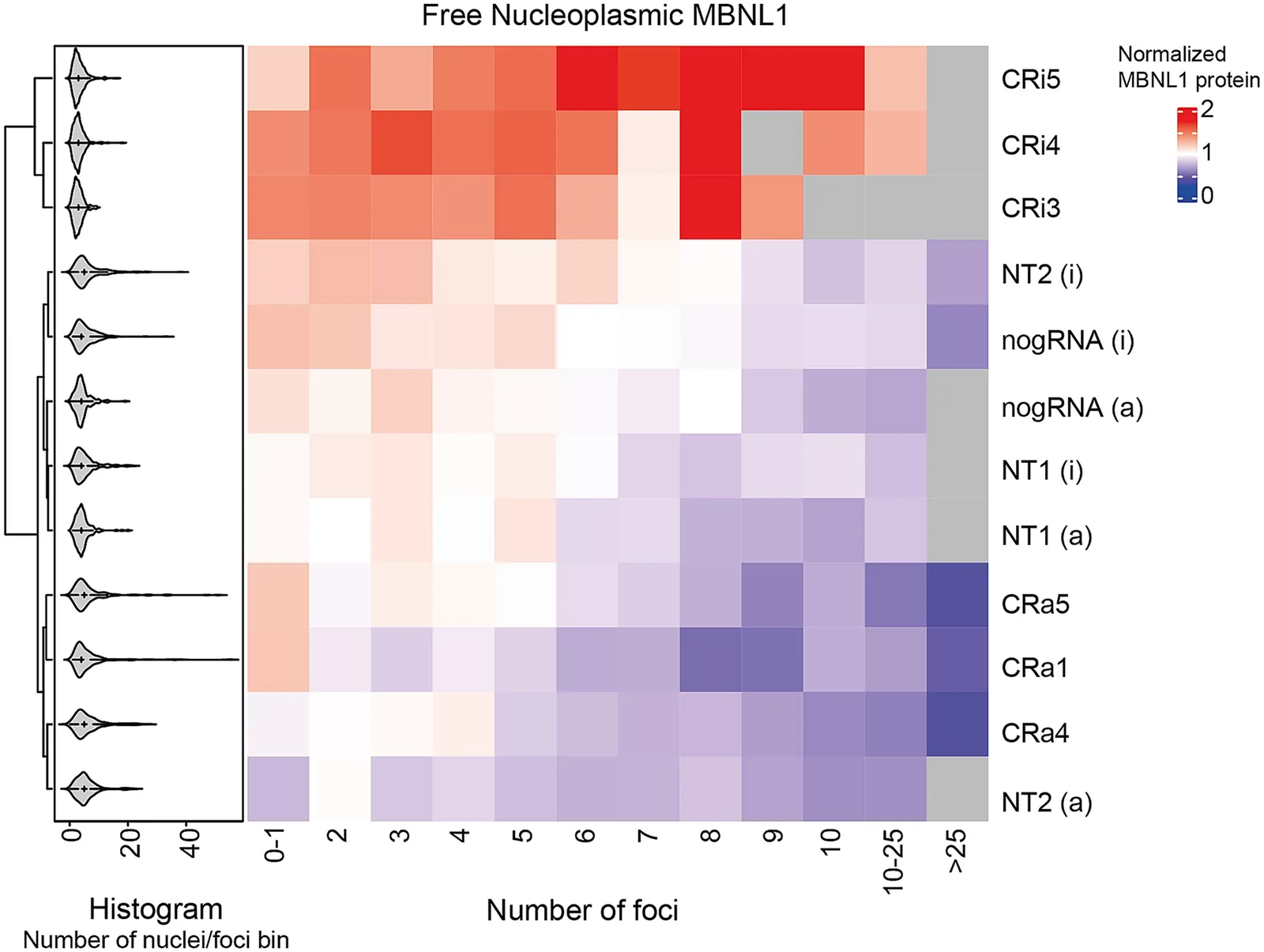
Linking single cell foci numbers to free nucleoplasmic MBNL1. DM1 cells treated with CRISPRi or CRISPRa as previously described were categorized per condition based on the amount of nuclear foci. Mean free nucleoplasmic MBNL1 (normalized to DM1 NT1) was calculated for each bin per condition and visualized in a clustered heatmap. The number of nuclei per bin is annotated in violin plots (left).

Collectively, our data demonstrate that expanded (CUG)n RNA levels determine nuclear MBNL1 availability, underscoring *DMPK* expression as a central determinant of DM1 molecular pathogenesis.

## Discussion

The complexity and heterogeneity of DM1 hinder a comprehensive understanding of its etiology, and, consequently, the development of effective therapeutic interventions. To gain mechanistic insight into the molecular drivers of disease manifestation, we isolated and studied the impact of expanded *DMPK* RNA levels on MBNL1 abundance, a key pathogenic factor in DM1, as well as on alternative splicing. Using the CRISPRa/i technique, we bidirectionally manipulated *DMPK* transcription to generate a controlled range of (CUG)2,900-transcript levels in isogenic human immortalized myoblasts. Variations in *DMPK* transcript abundance altered the pool of free nucleoplasmic MBNL1 and significantly affected established alternative splicing biomarkers. Collectively, these findings identify *DMPK* expression levels as a critical and quantitative determinant of repeat RNA disease burden and may adjust the design and efficacy of therapeutic approaches.

CRISPRi/a-mediated modulation of *DMPK* RNA levels likely affected both alleles: the expanded allele, as evidenced by changes in RNA foci number, and the non-expanded allele, as indicated by the observed CRISPRi-mediated reduction of total *DMPK* RNA by >80%. Although allele-specific targeting would be preferable for dissecting repeat-associated transcript toxicity, this approach was not feasible with CRISPR/dCas-based approaches because allele-specific SNPs are rare within the 500-bp region upstream and downstream of the *DMPK* transcription start site, and sgRNA design was limited by PAM-site availability and predicted on-target efficiency. We therefore took particular care to include the DM∆ control line, demonstrating that modulation of *DMPK* RNA levels in absence of repeat expansion had no effect on alternative splicing. Consistent with this, previous studies have shown that *DMPK* loss results in minimal or only mild, late-onset skeletal or cardiac muscle phenotypes in mouse and primate models [39–41], and antisense therapies currently evaluated in clinical trials likewise aim to block or degrade *DMPK* RNA [42–44]. Finally, CRISPRa/i off-target effects are unlikely, given the non-editing nature of dCas9 and low predicted off-target binding for the sgRNAs used.

CRISPRa increased *DMPK* RNA levels by up to 3.5-fold in DM1 myoblasts, but RNA foci numbers were heterogeneous with a significant increase observed in only a subset of cells. This variability may indicate that *DMPK* transcription may not be equally responsive to CRISPRa across all cellular states or cell-cycle phases. Compared with DM∆ controls, DM1 myoblasts required higher sgRNA concentrations for a measurable effect, suggesting that elevated levels of expanded *DMPK* RNA may actually exceed cellular tolerance thresholds. This apparent toxicity may arise from impaired stress responses resulting from dysregulation of stress granules and processing bodies, as previously reported in DM1 [45, 46]. These ribonucleoprotein assemblies play essential roles in RNA stability and are thought to form through liquid-liquid phase separation. Excessive CRISPRa-induced bursts of expanded *DMPK* RNA transcription could locally promote condensate formation of (CUG)n-expanded transcripts, consistent with recent observations in untreated DM1 cells [47]. Future studies employing inducible CRISPRa systems may help further elucidate the relationship between trinucleotide repeat expansion, RNA condensate dynamics and cellular stress responses.

CRISPRa-mediated increases in *DMPK* RNA expression exacerbated *MBNL1* missplicing, but had only limited effects on splicing of *NFIX* or *CLASP1*. This likely reflects the already severe basal splicing defects present in DM1 myoblasts and a consequently restricted dynamic range for further splicing alterations. Because alternative splicing is regulated by a tightly orchestrated network of splicing factors and RNA-binding proteins, we cannot rule out the possibility that compensatory mechanisms limited more extensive missplicing. Moreover, CELF1 phosphorylation was not altered in myoblasts lacking the repeat [36], suggesting that major changes in CELF1 activity did not contribute to the observed effects. Finally, the Ψ values reported for *MBNL1, NFIX* and *CLASP1* in these DM1 cell lines are comparable to those observed in the most severely affected MBNL1 knockout mouse models [24], consistent with the pronounced disease severity associated with a 2 900 CTG repeat expansion [32, 35].

CRISPRi resulted in efficient *DMPK* knockdown by up to 85% and restored free nucleoplasmic MBNL1 protein levels to those observed in DM∆ cells. Notably, however, missplicing of *MBNL1* and *NFIX* was not fully corrected. This incomplete rescue may be explained by the limited experimental time frame (48 h), but we consider this unlikely as *DMPK* downregulation was already evident 24 h after transfection (unpublished data), and foci numbers were decreased. In line with our observations, complementary studies to ours have been reported using CRISPRi, where this approach was proposed as a therapeutic intervention [48]. Taken together, a more plausible explanation lies in the dose-dependent model of MBNL1 activity, in which splicing outcomes are governed by graded MBNL1 availability and individual exons in different transcripts display distinct sensitivity thresholds [22]. In our study, we intervened upstream in the pathogenic cascade by modulating transcription of the expanded *DMPK* allele. Downregulation of a highly expanded *DMPK* allele, representative of severe DM1, fully rescued *CLASP1* splicing, in line with predictions from the model proposed by Wagner et al. [22]. However, *NFIX*, a biomarker associated with milder DM1 and intermediate MBNL1 protein levels, was only partially rescued. Thus, while CRISPRi effectively attenuated severe DM1-associated splicing defects, achieving correction of more sensitive splicing events will likely require even more extensive reduction of expanded *DMPK* RNA levels.

Reduction of expanded *DMPK* RNA clearly resulted in an ameliorated phenotype, as shown by improvements in MBNL1-mediated alternative splicing. However, this leaves a key unresolved question: How much reduction is required to achieve a clinically meaningful benefit in a clinical trial in DM1 patients? We hypothesize that moderate downregulation of expanded *DMPK* transcripts, as achieved here using CRISPRi, may already confer a significant therapeutic effect, consistent with outcomes reported for antisense oligonucleotide-base strategies [49, 50]. Nevertheless, more profound knockdown levels, exceeding 80%, may be necessary for complete reversal of disease manifestations, likely in a cell-, tissue- and organ-dependent manner. Such therapeutic thresholds are expected to correlate with intrinsic *MBNL1* expression levels and local repeat length, which is further shaped by ongoing somatic expansion [51].

Future studies applying CRISPRi/a technology to myoblasts harboring short and intermediate CTG repeat lengths may help elucidate the precise relationship between RNA abundance, repeat length, MBNL1 availability, and splicing dysregulation. One can envisage that modulation of expanded *DMPK* RNA at shorter repeat lengths would induce smaller, more graded changes in free MBNL1 activity compared with the pronounced effects observed in the myoblasts carrying 2,900 triplets here, even though baseline missplicing will be less severe. Further, investigating the effects of *DMPK* modulation during myogenic differentiation would be of considerable interest, as both *DMPK* and *MBNL* are dynamically and differentially regulated throughout this process, particularly during myoblast fusion and the early stages of muscle fiber formation [52, 53]. Consequently, the molecular and phenotypic consequences of tuning *DMPK* expression are likely to be strongly dependent on the stage of differentiation. Finally, given that DM1 is a multisystemic disease, studies in other disease-relevant cell types are warranted, as the precise interplay between *DMPK* expression and MBNL1 protein abundance is likely cell-type dependent.

In conclusion, our findings demonstrate that expanded *DMPK* RNA levels act as a quantitative driver of DM1 pathogenesis, governing MBNL1 availability and downstream splicing outcomes in a dose-dependent manner. Future studies enabling finer temporal and quantitative control of *DMPK* expression will be essential to define therapeutic thresholds and optimize RNA-targeting strategies in DM1 patients.

## Materials and Methods

### Cell culture

Human immortalized DM1 myoblasts expressing an expanded *DMPK* gene with around 2,900 triplets were propagated in proliferation medium, i.e. a 1:1 mix of Skeletal Muscle Growth Medium (PromoCell, Heidelberg, Germany) and Ham's F10 nutrient mix (Gibco, Thermo Fisher Scientific, Landsmeer, the Netherlands), supplemented with 20% (v/v) Hyclone Bovine Growth Serum (Cytiva, Medemblik, the Netherlands) and GlutaMax (Gibco) in adherent culture flasks, coated with 0.1% gelatin (G2500; Sigma-Aldrich, Merck KGaA, Darmstadt, Germany) [32, 35, 37]. Cell confluency was kept below 80% to avoid myogenic differentiation. Cultures were refreshed every 2-3 days and incubation was performed at 37°C and 7.5% CO_2_ under humidified conditions.

### Lentiviral transduction

The lentiviral constructs used to generate myoblasts stably expressing dCas9-VPR or dCas9-KRAB were kind gifts from John Doench & William Hahn & David Root (Addgene plasmid #96918; and Addgene plasmid #96917) [38, 54]. Myoblast cell lines were stabilized using lentiviruses produced by the Forward Transfection Procedure of the ViraPowerTM Lentiviral Expression system (Thermo Fisher Scientific, Breda, Netherlands). In short, HEK293FT cells were cultured in DMEM high glucose medium (Gibco) supplemented with 1% sodium pyruvate, 1% GlutaMax (Gibco), 1% penicillin/streptomycin (Gibco) and 10% fetal calf serum (Sigma-Aldrich). The cell lines were cultured in a humidified chamber at 37°C and 5% CO_2_. Myoblast cell lines were transduced with lentivirus-containing medium directly harvested from the HEK293FT cultures plus 0.1% polybrene (Sigma-Aldrich). After 8 hrs, the medium was replaced by myoblast proliferation medium. Blasticidin selection (10ug/mL) was started after 3 days to generate stable expressing cells. All lines were a pool of stable cells, except the DM1 line expressing dCas9-KRAB, which was generated based on a single cell clone.

### Transfection synthetic sgRNA

Short guide RNAs for CRISPRa/i were designed using CRISPR-ERA (CRISPR-ERA (stanford.edu)), GPP sgRNA designer (Broad Institute) and checked for predicted off-target effects using the gRNA tools of IDT, dna20 and Chopchop. Verified non-targeting sgRNA sequences were derived from Sigma. All synthetic sgRNAs were ordered as crRNA + tracrRNA in one and were stabilized by including 2’-O-methyl and phosphorothioate modifications. Sequences and target strand are displayed in Supplementary Table 1.

Prior to transfection, myoblasts were seeded at 10,000-15,000 cells/cm^2^ in 0.1% gelatin (Sigma)-coated 12-wells plates in which optionally also a 12-mm coverslip (EMS) was included for microscopy. Cells were left to adhere for 4 hours in the incubator. Transfection of sgRNA was performed using Lipofectamine 3000 (Thermo Fisher Scientific) according to manufacturer's protocol. For a single well, the required amount of sgRNA to reach the final sgRNA concentration was mixed with 50 μL Opti-Mem (Gibco). In parallel, 3 μL of Lipofectamine 3000 was mixed with 50 μL of Opti-Mem. These two solutions were mixed 1:1 and left to incubate for 10 min at RT before being added to the cell cultures. Two wells per condition were used as technical replicates. After 48 hours, cell cultures were washed with PBS. Coverslips were removed from the well and transferred to 2% PFA in 0.1 M phosphate buffer pH 7.4 to fixate. Lysis for RNA isolation was subsequently performed on the remaining cells present in the well.

### RNA isolation and RT-(q)PCR

RNA was isolated using the Aurum Total RNA mini kit (Bio-Rad Laboratories, Veenendaal, the Netherlands) according to manufacturer's protocol. Prior to the ethanol step, lysates were pulled through a 0.4 mm syringe 12 times to ensure DNA shearing. DNase I treatment was performed on the column as recommended by the manufacturer. Concentration and quality of RNA was measured using 260/280 and 260/230 absorption on the NanoVue Plus Spectrophotometer (GE Healthcare Life Sciences). cDNA was made using iScript cDNA synthesis kit (Bio-Rad) with 500 ng RNA input or the maximal allowed volume of 15 μL RNA sample. *DMPK* expression was subsequently determined by RT-qPCR using two *DMPK* primer pairs and reference genes *GAPDH* and *HPRT1* (primer sequences are shown in Supplementary Table 2) [55]. RT-qPCR was run in a mixture consisting of 5 μL iQ SYBR Green supermix (Bio-Rad), 0.4 μM forward and reverse primers, 3 μL of 10-fold diluted cDNA and 0.4 μL of mQ on a CFX96 Touch Real-Time PCR Detection System (Bio-Rad). RT- and NTC were taken along for each replicate. Normalization was performed using the 2^−ΔΔCt^ method and values of NT1 in the corrected cell line was set at 1.

### Splicing analysis

To assess alternative splicing for selected cassette exons, RT-PCRs were run using a mixture of 0.4 U Q5 High-fidelity DNA polymerase (New England Biolabs (NEB), Leiden, the Netherlands), 0.5 μM forward and reverse primer each (see Supplementary Table 2 for sequences [22]), 1x Q5 reaction buffer (NEB) and 0.2 mM dNTPs (Thermo Fisher Scientific). 4-8 μL input of 10-fold diluted cDNA was used as input, UltraPure distilled Water (Invitrogen, Carlsbad, CA, USA) was used to fill up the reaction volume to 20 μL. The number of cycles were kept limited to avoid PCR mix exhaustion and therefore skewing towards smaller amplicons. Samples were subsequently analyzed via the QIAxcel Advanced capillary electrophoresis system (Qiagen, Germantown, MD, USA) using the DNA high resolution cartridge, 15-600 bp alignment marker and 25-500 bp size marker (Qiagen). Quantitative analyses for product concentration were conducted using the QIAxcel ScreenGel Software (Version 1.5.016). The peak calling function was used to determine molarities for all amplicons. PSI values were calculated by dividing the molarity of the amplicon of the cassette exon over the total molarity.

### Immunofluorescence assays

To assess RNA foci number and MBNL1 protein abundance, cells were fixed 48 hours after sgRNA transfection. FISH was performed using the Stellaris RNA FISH components and protocol for adherent cells (LGC Biosearch Technologies, Petaluma, CA). In short, cells were washed once with ethanol and permeabilized in 70% (v/v) ice-cold ethanol for a minimum of one hour at 4°C and washed in Stellaris Wash Buffer A, before hybridization with 12.5 ng/mL probe CAG_6_-alexa647 (Eurogentec S.A., Seraing, Belgium) in hybridization buffer at 37°C for 16 hrs. Cells were washed once more with Wash Buffer A for 30 minutes at 37 °C and finished in Wash Buffer B. Next, immunofluorescence analysis for MBNL1 was performed. Myoblasts were washed three times in PBS and permeabilized once again in 0.1% (v/v) Triton X-100 (Sigma-Aldrich) in blocking buffer consisting of 3% (w/v) bovine serum albumin and 0.1% (w/v) glycine (Sigma-Aldrich) in PBS for 5 min at RT. Cells were then blocked for 1 hr in blocking buffer. Overnight incubation with primary antibody (Developmental Studies Hybridoma Bank) in blocking buffer was performed at 4 °C. After washing three times with 1x PBS, samples were incubated with matching secondary antibodies and 1 ug/mL DAPI (Sigma-Aldrich) in blocking buffer for 1 hr at RT. Finally, all samples were washed three times with PBS and enclosed in Mowiol or Fluormount G. Imaging was performed blinded to sample identity using a Zeiss LSM900 with a 63x oil objective (NA 1.4) with imaging settings for DAPI (405 nm), AF488 and AF647. Z-stacks of 1 μm were made to image the entire cell nucleus.

### Microscopical analysis

To objectively quantify the microscopy data set, a macro script was written in FIJI ImageJ v1.53t. In short, z-stacks for all three channels were maximum projected based on maximum signal intensity. The DAPI signal was evened out (median filter), and a binary mask was created for each image using Auto Threshold Huang 2 and subsequent optional water shedding to separate close nuclei. A ROI was created per nucleus by the analyze particle tool. RNA foci were counted in the FISH channel using the find maxima tool. For MBNL1 abundance, total nuclear MBNL1 signal per nucleus and sequestered MBNL1 levels was measured in a 81px square seeded by the FISH signal. Free nucleoplasmic MBNL1 was calculated per individual nucleus by subtracting sequestered MBNL1 in all foci of total nuclear MBNL1. Normalization on sgRNA NT1 was performed per independent experiment, foci counts were kept as an absolute number.

### Statistics

All analyses were performed using Microsoft Excel and R using the R studio interface. Visualization was performed using the ggplot2 package with viridis color pallets. Figures addressing RNA results were based on three fully independent experiments in which cells were seeded on separate days and RNA was isolated according to the experimental timeline. Within each experiment, technical duplicates (i.e., two separately treated but identically handled wells) were typically included. Error bars represent standard error of the mean. Significance was tested using one-way ANOVA with, if p < 0.05 for the ANOVA test, Tukey post-hoc testing for significance between conditions. Microscopy data display single data points of three independent experiments, 5-10 images per condition per experiment. ^*^P < .05, ^**^P < .01, ^***^P < .001, ^****^P < .0001.

## Supplementary Material

Ripken_et_al_CRISPRa_i_SI_revision_final_clean_ddag079

## Data Availability

All data underlying this article are available within the article and its online supplementary material. Additional data, including results or materials not explicitly included or anticipated at the time of publication, may be provided by the corresponding author upon reasonable request.
