# Supplementary material for: CRISPR/dCas9-mediated tuning of DMPK transcription reveals a quantitative relationship between toxic repeat RNA expression and MBNL1 activity in myotonic dystrophy
Source: Hum Mol Genet. 2026 Aug 21;35(17):ddag079. doi: 10.1093/hmg/ddag079 (PMC13648012; doi:10.1093/hmg/ddag079)
Supplement: Supplementary file 1 [file ripken_et_al_crispra_i_si_revision_final_clean_ddag079.pdf]

Supplemental information

Supplemental figures and tables

Supplemental Table 1: Sequences of sgRNAs used in this study.

CRISPRi/a	sgRNA	gRNA sequence (5' -> 3')	Target strand
a and i	NT1	CGCGAUAGCGCGAAUAUAUU	n/a
a and i	NT2	GCGCGAUAGCGCGAAUAUAC	n/a
a	CRa1	GAACCCUUCAGUGCUAGAGU	+
a	CRa4	CUGGGUCACUCAGCCUCAAU	-
a	CRa5	GCUUGUCGGGAGCAAGGGGG	-
i	CRi3	GACAGGCAGACAUGCAGCCA	-
i	CRi4	CGGCCGGCCGCAGAGAGAAG	-
i	CRi5	CCUGUCCAACAUGUCAGCCG	-

Supplemental Table 2: PCR primers used in this study.

Gene	Forward sequence (5'-> 3')	Reverse sequence (5'-> 3')	Assay
DMPK e1-2	ACTGGCCCAGGACAAGTACG	CCTCCTTAAGCCTCACCACG	RT-qPCR
DMPK e15 3'	TGCCTGCTTACTCGGGAAATT	GAGCAGCGCAAGTGAGGAG	RT-qPCR
GAPDH	CCCGCTTCGCTCTCTGCTCC	CCTTCCCCATGGTGTCTGAGCG	RT-qPCR
HPRT1	TGACACTGGCAAAACAATGCA	GGTCCTTTTCACCAGCAAGCT	RT-qPCR
MBNL1	AGGGAGATGCTCTCGGGAAAAGTG	GTTGGCTAGAGCCTGTTGGTATTGGAAAATAC	Splicing
NFIX	GATGGAGAGCCCTGTTGATGACG	GTGGTGGTGGTAGCGGATGGTC	Splicing
CLASP1	CAAAGTCTCCTCATCTTCGGGCACG	GCTGGGACTGTGAAACCACTTTAGC	Splicing

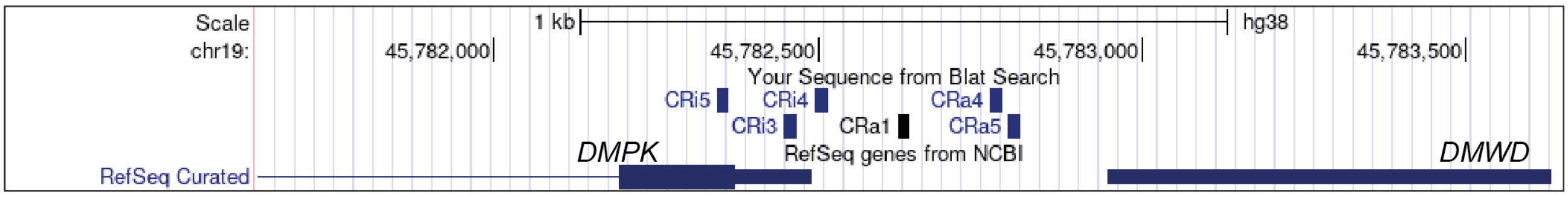

Supplemental Figure 1: Location of gRNAs in regard to *DMPK* and *DMWD.*

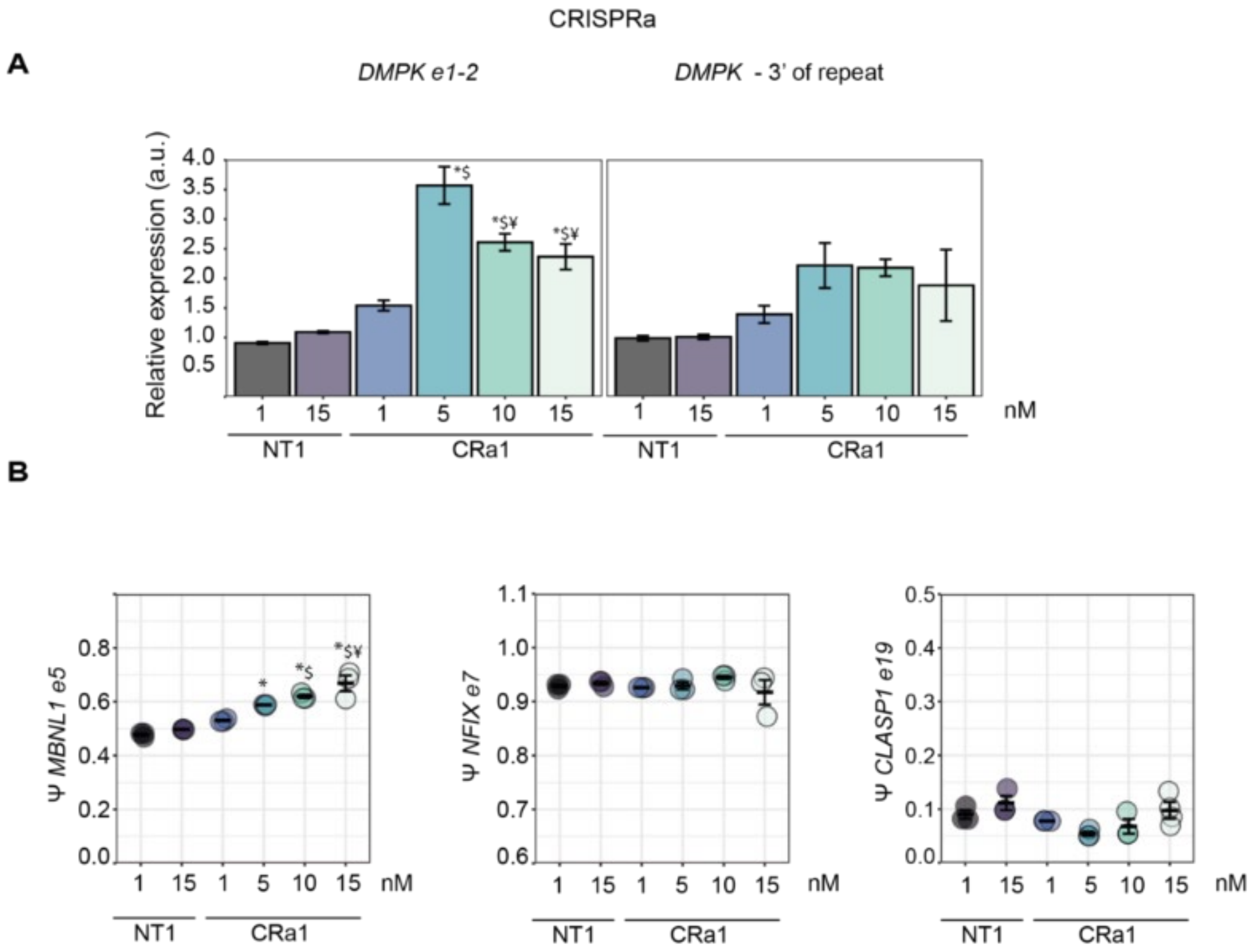

Supplemental Figure 2: Increased *DMPK* transcript numbers aggravate *MBNL1* splicing in a sgRNA dose-dependent manner. $DM1^{dCas9VPR}$ myoblasts were transfected with incrementing sgRNA concentrations of sgRNA CRa1. After 48 hours, RNA was isolated to assess *DMPK* RNA levels (A) or alternative splicing of DM1 biomarkers (B). One-way ANOVA was performed to test for significance. n=3 independent experiments, with two wells per condition per experiment as technical duplicate. Error bars depict SEM. * $p<0.01$ compared to NT1 1 nM and NT1 15 nM, $ $p<0.05$ compared to CRa1 1 nM, ¥ $p<0.05$ compared to CRa1 5 nM

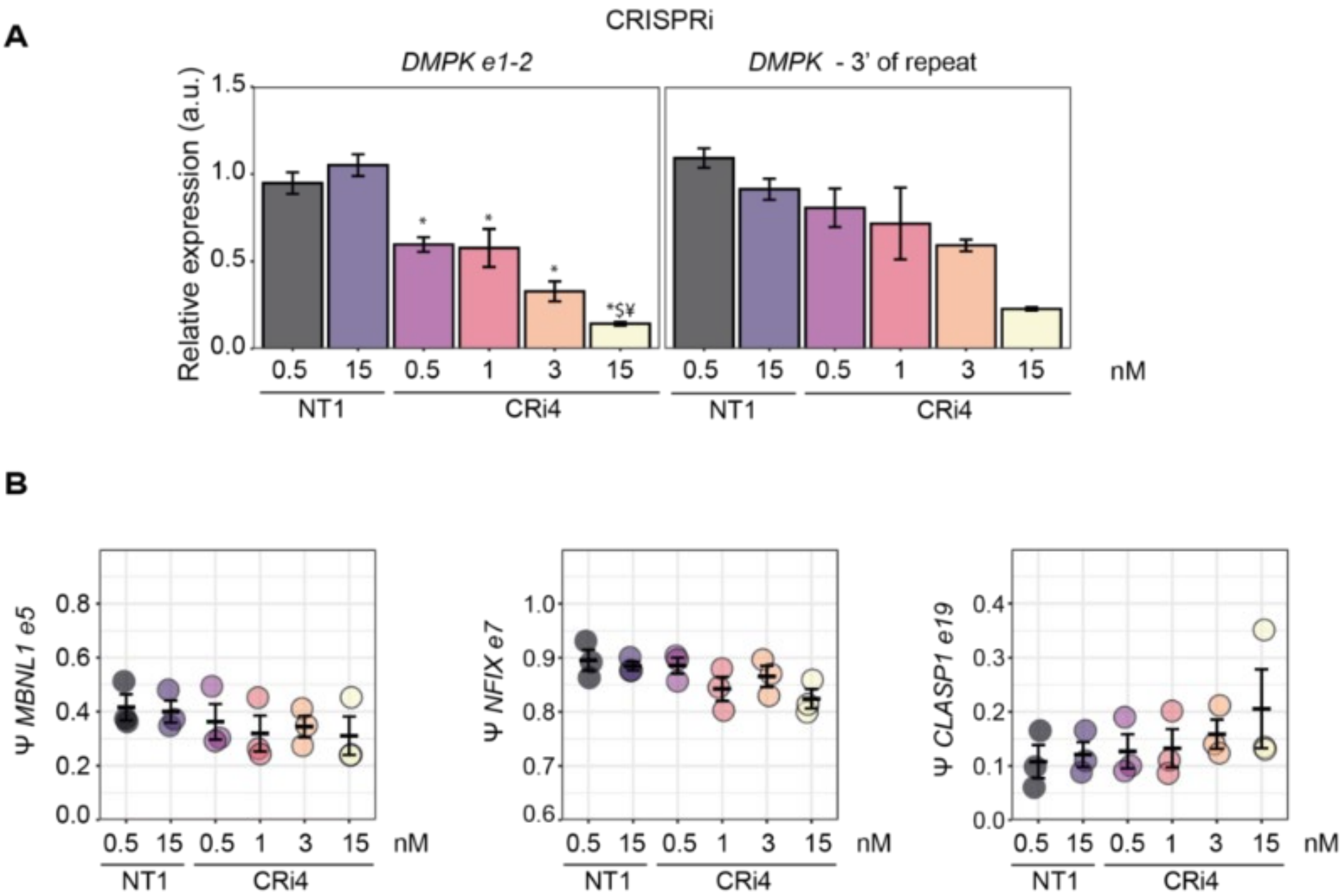

Supplemental Figure 3: Dose-dependent downregulation of *DMPK* RNA using CRISPRi. DM1^{dCas9KRAB} myoblasts were transfected with incrementing sgRNA concentrations of sgRNA CRi4. After 48 hours, RNA was isolated to assess *DMPK* RNA levels (A) or alternative splicing of DM1 biomarkers (B). One-way ANOVA was performed to test for significance. n=3 independent experiments, with two wells per condition per experiment as technical duplicate. Error bars depict SEM. * p<0.01 compared to NT1 0.5 nM and NT1 15 nM, $ p<0.05 compared to CRi4 0.5 nM, ¥ p<0.05 compared to CRi4 1 nM.
